# Severe eosinophilic pneumonia presenting during gemcitabine adjuvant chemotherapy

**DOI:** 10.1186/1477-7819-11-167

**Published:** 2013-07-24

**Authors:** Tomomi Yakabe, Kenji Kitahara, Kazutoshi Komiya, Naoko Sueoka-Aragane, Shinya Kimura, Takashi Sugioka, Hirokazu Noshiro

**Affiliations:** 1 Department of Community Medical Support Institute, Saga University, Saga, Japan; 2 Department of Surgery, Saga University Faculty of Medicine, Saga, Japan; 3 Division of Hematology, Respiratory Medicine and Oncology, Department of Internal Medicine, Faculty of Medicine, Saga University, Saga, Japan

**Keywords:** Pancreatic cancer, Gemcitabine, Adjuvant chemotherapy, Eosinophilic pneumonia, Acute lung injury, Adverse event, Pulmonary toxicity

## Abstract

Gemcitabine is widely accepted as the standard treatment for pancreatic cancer, but it can cause unpredictable side effects. Acute respiratory distress syndrome is a rare complication with gemcitabine, but is sometimes fatal. We describe a cured case of acute, severe gemcitabine-induced pulmonary toxicity. The patient was a 76-year-old man with pancreatic cancer who was receiving adjuvant gemcitabine chemotherapy after surgery. The patient received gemcitabine 1,000 mg/m^2^ on days 1, 8, and 15 for three 4-week cycles, with intervals of 1 week. He developed severe general fatigue on day 1 of the third cycle. Computed tomography showed diffuse ground-glass opacity with pleural effusion. There was no increase in β-D-glucan, and cytomegalovirus antigenemia assays were negative. No bacteria or acid-fast bacilli were found. The number of eosinophils in bronchoalveolar lavage fluid was increased. Considering these data, we diagnosed eosinophilic pneumonia induced by gemcitabine. The patient was immediately treated with a steroid and neutrophil elastase inhibitor under respiratory supportive therapy. After 4 weeks, his pulmonary symptoms were markedly improved. Physicians should be cognizant of the possible association of serious pulmonary toxicity with gemcitabine treatment. A delay in diagnosis and treatment could lead to a fatal outcome.

## Background

Surgery is considered to be only curative option for patients with resectable pancreatic cancer. However, adjuvant therapy is considered to improve the surgical outcome because there is the high recurrence rate of the disease even after curative resection [1-4]. Gemcitabine (2’,2’-difluoro-s’-deoxycytidine) is widely accepted as the standard treatment for pancreatic cancer.

Two recent randomized phase III trials of adjuvant chemotherapy have been conducted in patients with resected pancreatic cancer. The CONKO-001 study comparing surgery followed by gemcitabine with surgery alone suggested that adjuvant gemcitabine did indeed contribute to prolongation of disease-free survival, although overall survival did not differ significantly between the two groups [1]. The JSAP-02 study undertaken in Japan showed similar results [5], differing only in the planned number of gemcitabine cycles: three cycles of gemcitabine were used in JSAP-02 in contrast to six cycles in CONKO-001. The most common side effect of gemcitabine is myelosuppression. Severe pulmonary toxicity as a side effect is relatively rare, with a reported incidence of between 0 and 5%, but 20% of patients with severe pulmonary toxicity died [6].

We describe a rare case of potentially fatal severe eosinophilic pneumonia induced by gemcitabine, which was successfully treated.

## Case presentation

The patient was a 76-year-old manreceiving gemcitabine chemotherapy for stage IIIA pancreatic cancer after undergoing subtotal stomach-preserving pancreaticoduodenectomy for a tubular adenocarcinoma in the pancreas. Six weeks later, the patients was started on gemcitabine (Gemzar®; Eli Lilly, Indianapolis, IN, USA) at a dose of 1000 mg/m^2^ over 30 min on days 1, 8, and 15 every 4 weeks. This 4-week cycle was repeated for three cycles, with a 1-week interval between them, based on the protocol used in the JSAP-02 study [5].

The patient developed severe general fatigue on day 1 of the third cycle. Arterial blood gas analysis showed hypoxemia (PaO_2_ 62 Torr with 8 l/min of oxygen by face mask). His white blood cell count was 17.1 × 10^9^/l with 80.7% neutrophils and 1.0% eosinophils. He reported general fatigue and dyspnea but no cough.

**Figure 1:**
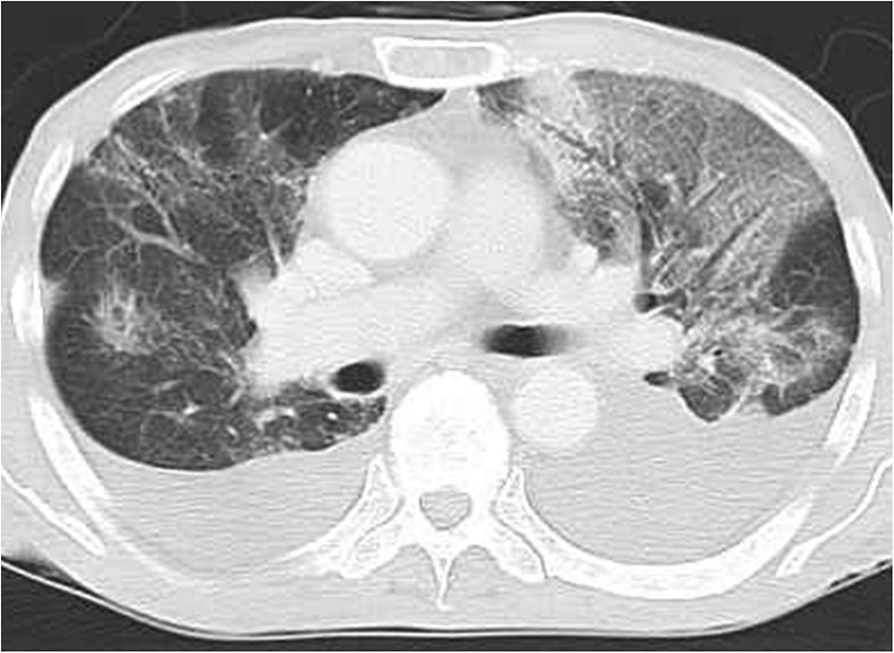
**Chest computed tomography on admission.** Bilateral pleural effusion and diffuse ground-glass opacity was apparent, and thickened septal lines and diffuse reticular opacity were evident in both lungs.

**Figure 2:**
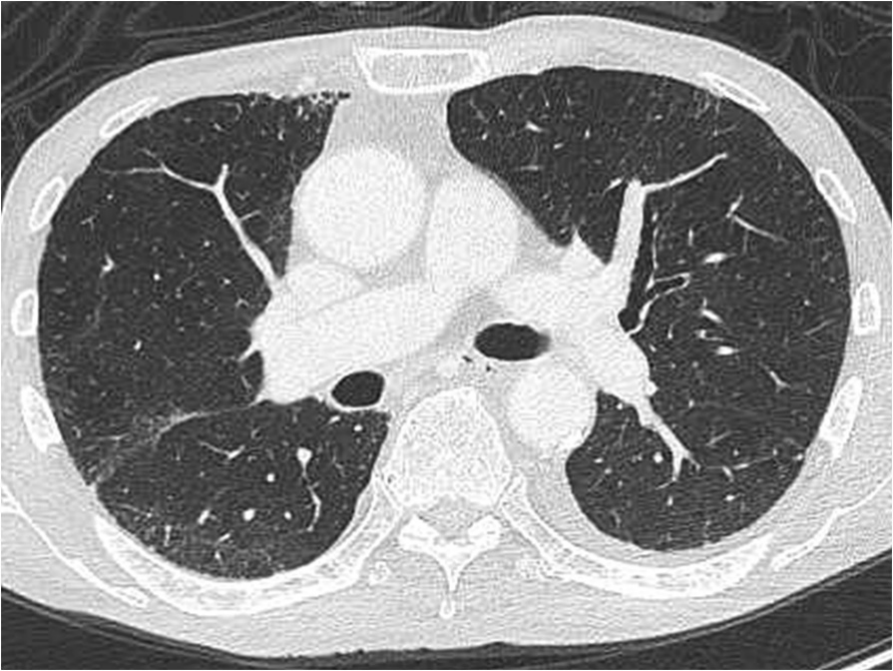
**Chest computed tomography at 4 weeks after starting treatment.** The ground-glass opacity in both lungs disappeared after discontinuation of gemcitabine and initiation of corticosteroids and pulmonary support.

On physical examination, the patient had negligible crackles and poor inspiration in both lungs. Computed tomography (CT) showed pleural effusion in both sides of the thorax, and diffuse ground-glass opacity, thickened septal line,s and diffuse reticular opacity in both lungs (Figure 1). There was no increase in β-D-glucan, and cytomegalovirus antigenemia assays were negative. No bacteria or acid-fast bacilli were found. Pleural fluid cytology was also negative for cancer cells. Laboratory studies revealed that the patient’s lactate dehydrogenase level was 253 U/l, KL-6 was 298 U/ml, and SP-D was 107 ng/ml. Antibiotics and sivelestat sodium hydrate had been started on admission, under a working diagnosis of acute lung injury due to infection. However, the patient’s respiratory status did not improve. Bronchoalveolar lavage (BAL) was performed for further differential diagnosis. The percentage of eosinophils in the BAL fluid was raised (56%), strongly suggesting a diagnosis of gemcitabine-induced acute eosinophilic pneumonia. The patient was treated with methylprednisolone 1000 mg/day and sivelestat sodium hydrate for 3 days, with methylprednisolone 40 mg/day for a further 11 days, and then started on bi-level positive airway pressure (BiPAP Vision®; Philips International, Amsterdam, The Netherlands) by face mask to provide non-invasive pressure support at 5 cm H_2_0. After 4 weeks, there was marked improvement in the pulmonary symptoms and CT findings (Figure 2).

Gemcitabine is relatively well tolerated, and myelosuppression is generally the dose-limiting toxicity; pulmonary toxicity with gemcitabine is relatively uncommon. The incidence of grade 4 lung toxicity ranges from 0.06%, as reported by an industry study based on commercial exposure, to 8%, as reported in several case study reviews, and the mortality rate is 20% [7,8]. In addition, gemcitabine-induced eosinophilic pneumonia has been reported previously [9,10]. Thus, diagnosis of drug-induced pneumonia has now become more important for patients with cancer. We also investigated the prevalence of drug-induced pneumonia in patients with pancreatic cancer or biliary tract cancer. From data collected on 92 patients treated with gemcitabine alone for pancreatic or biliary tract cancer between January 2007 and December 2010 at our institution (Table 1), the common reasons for treatment delay were myelotoxic effects, infection, and emesis. Discontinuation because of grade 3 or 4 myelosuppression, hepatobiliary disorder, and malaise occurred in 4 (4.0%) of 100 events. Drug-induced pneumonia as the main reason for discontinuation accounted for two of six cases (33.3%) in whom gemcitabine was discontinued, suggesting that gemcitabine-induced pneumonia has significant morbidity and mortality.

**Table 1:** Reasons for delay or discontinuationin patients who received gemcitabine systemic chemotherapy

	**No. of patients (%)**	
Non-hematological, n (%)	Treatment delay, n = 94^a^	Discontinuation of gemcitabine, n = 6
Vomiting	5 (5.3)	0
Infection	10 (10.6)	0
Drug-induced pneumonia	0	2 (33.3)
Diarrhea	2 (2.1)	0
Malaise	4 (4.3)	1 (16.7)
Patient’s wish	3 (3.2)	0
Hematological, n (%)		
Neutrophil count decreased	36 (38.3)	0
Anemia	7 (7.4)	1 (16.7)
Platelet count decreased	15 (16.0)	1 (16.7)
AST or ALT increased	4 (4.3)	0
Blood bilirubin increased	5 (5.3)	1 (16.7)
Creatinine increased	1 (1.1)	0
Hypoalbuminemia	2 (2.1)	0

Abbreviations: *ALT*, Alanine aminotransferase; *AST*, Aspartate aminotransferase.

^a^Total exceeds 92 because of the duplication of patients.

Pulmonary eosinophilic infiltrates are a heterogeneous group of disorders characterized by the presence of eosinophils in the lungs, as detected by BAL or tissue biopsy, with or without blood eosinophilia. Pulmonary infiltrates, characterized by foci of air-space consolidation and focal ground-glass opacity, can be seen in pulmonary eosinophilia of all causes [11]. The findings from CT and careful clinical evaluation must correspond in order for a definitive diagnosis to be made. BAL is the principal method of confirming the diagnosis of acute eosinophilic pneumonia. In such cases, eosinophils account for more than 25% of the cells in the BAL fluid [12]. In the present case, eosinophils showed an increase to 56% on BAL analysis, and we ultimately used this finding to reach the diagnosis of eosinophilic pneumonia.

Once the diagnosis has been made, the most common treatment involves stopping gemcitabine and starting high-dose steroids such as hydrocortisone, methylprednisolone, and dexamethasone, which are then tapered over the course of several weeks alongside oxygen supplementation. In the present case, treatment was started using sivelestat sodium hydrate (ONO-5046), a neutrophil elastase inhibitor developed in Japan. Neutrophil elastase is a protease produced by inflammatory cells, and its inhibition is associated with the prevention of acute lung injury (ALI) in animal models [13]. It has been suggested that the physiopathological mechanism of drug-induced ALI is a cytokine-mediated inflammatory reaction of the alveolar capillary wall [14]. Although the importance of leukocyte elastase relative to other proteases in the pathogenesis of lung injury remains uncertain, we selected this drug on the expectation of beneficial effects of elastase inhibition for drug-induced ALI.

## Conclusion

We emphasize that physicians must be cognizant that of the possible association of serious pulmonary toxicity with gemcitabine treatment. A delay in diagnosis and treatment of gemcitabine-induced severe eosinophilic pneumonia could lead to a fatal outcome.

## Consent

Written informed consent was obtained from the patient’s next of kin for publication of this Case report and any accompanying images. A copy of the written consent is available for review by the Editor-in-Chief of this journal.

## Abbreviations

ALI: Acute lung injury; ALT: Alanine aminotransferase; AST: Aspartate aminotransferase; BAL: Bronchoalveolar lavage; CT: Computed tomography; KL-6: Krebs von den Lungen-6; SP-D: Surfactant, pulmonary-associated protein D.

## Competing interests

The authors declare that they have no competing interests.

## Authors’ contributions

TY drafted the manuscript and made revisions. KK performed the surgery. KK and NA participated in the medical treatment. SK and TS helped to draft the manuscript. HN was lead surgeon and reviewed the manuscript. All authors read and approved the final manuscript.
